# RAF/MEK clamp inhibition with avutometinib and cetuximab in RAF1 S257L–mutated, anti-EGFR–refractory metastatic colorectal cancer in Gaucher’s disease

**DOI:** 10.1093/oncolo/oyag335

**Published:** 2026-08-19

**Authors:** Ashley Rose, Alix Rosenberg, Udhayvir Singh Grewal, Timothy J Brown, Nicholas Hornstein

**Affiliations:** Lenox Hill Hospital, New York, NY 10075, United States; Northwell Health Cancer Center, New York, NY 11042, United States; Donald and Barbara Zucker School of Medicine at Hofstra University, New York, NY 11549, United States; Department of Hematology Oncology, Winship Cancer Institute of Emory University, Atlanta, GA 30322, United States; Department of Hematology Oncology, UT Southwestern Medical Center, Dallas, TX 75390, United States; Northwell Health Cancer Center, New York, NY 11042, United States

**Keywords:** RAF/MEK, avutometinib, RAF1, colorectal cancer, Gaucher disease

## Abstract

*RAF1* alterations are rare in metastatic colorectal cancer (mCRC), occurring in roughly 0.5% of cases, and lack established treatment guidance. We present a patient with *RAF1* S257L–mutated, RAS wild-type mCRC and concurrent type 1 Gaucher disease, whose baseline cytopenias precluded standard chemotherapy and clinical-trial enrollment. After progression on fluoropyrimidine-, oxaliplatin-, and irinotecan-based regimens (the latter with the anti-EGFR antibody panitumumab) complicated by recurrent neutropenic sepsis, he received off-label avutometinib and defactinib (an oral RAF/MEK clamp inhibitor plus a FAK inhibitor) combined with cetuximab. He achieved a radiographic response, biomarker decline, hematologic recovery, and improvement in ECOG performance status from 3 to 0, with benefit lasting approximately 6 months. At progression, circulating tumor DNA revealed persistence of *RAF1* S257L at high allele fraction with *RAF1* amplification, together with newly acquired EGFR ectodomain mutations, *KRAS* alterations, and a *MAP2K1* alteration—a polyclonal pattern of convergent *MAPK/EGFR* reactivation. This case illustrates how a molecular tumor board can translate a rare driver into a rational, individualized regimen and how serial liquid biopsy informs resistance and next steps.

Key Points

- *RAF1* S257L is a rare, activating MAPK-pathway alteration in colorectal cancer that constitutively drives signaling and is a recognized mechanism of resistance to anti-EGFR monotherapy; anti-EGFR antibodies alone are therefore not expected to be effective.
- A RAF/MEK clamp inhibitor (avutometinib) combined with cetuximab produced a radiographic response and major functional recovery in a heavily pretreated, anti-EGFR–refractory patient, suggesting RAF/MEK pathway blockade—not EGFR blockade alone—drove the benefit.
- Comorbidities matter: Gaucher disease amplified chemotherapy-related cytopenias and excluded trial participation, making a better-tolerated targeted regimen the pragmatic choice.
- Serial circulating tumor DNA at progression showed convergent MAPK/EGFR reactivation (acquired EGFR ectodomain and *KRAS* alterations, *RAF1* amplification, and *MAP2K1*), consistent with on-pathway resistance and informing subsequent therapy.
- Serial liquid biopsy is a practical tool when tissue is limited and patients are too frail for repeated invasive procedures. Here, it established the baseline driver, tracked treatment response alongside CEA, and characterized the key resistance mechanisms at progression.

## Patient story

A man in his late 50 s with type 1 Gaucher disease^1^ presented with abdominal pain and severe constipation and was found to have a perforated sigmoid colon. He underwent emergency resection with pathology demonstrating moderately differentiated, microsatellite-stable adenocarcinoma (7 of 21 lymph nodes positive, three tumor deposits, perineural and lymphovascular invasion) with peritoneal involvement and radiographic hepatic metastases (stage IV). His postoperative course was prolonged and complicated by septic shock, deconditioning, and cytopenias related to Gaucher disease. At the time of the medical oncology assessment, his ECOG performance status was 3.

Tumor molecular profiling with NGS showed *KRAS/NRAS* wild-type status with an *APC* mutation, a *TP53* mutation, a *RAF1* S257L pathogenic variant, and a homozygous UGT1A1*28/*28 genotype. After rehabilitation he began 5-fluorouracil, escalated to 5-fluorouracil and oxaliplatin (modified FOLFOX), with bevacizumab added; oxaliplatin was subsequently held for thrombocytopenia after cycle four, and enzyme replacement therapy with imiglucerase was started for Gaucher’s disease. His cancer progressed, resulting in a bowel obstruction and rising carcinoembryonic antigen (CEA) after cycle four. This prompted a switch to dose-reduced 5-fluorouracil with irinotecan (modified FOLFIRI) with panitumumab. This was again complicated by severe neutropenia (absolute neutrophil count nadir ∼0.16 × 10^9^/L), neutropenic fever, recurrent pancytopenia, and septic shock refractory to growth-factor support after cycle two; as a result, his cancer progressed on this anti-EGFR–containing regimen. With no tolerable standard options and trial ineligibility due to persistent cytopenias, his case was brought to the molecular tumor board.

## Molecular tumor board

### Genotyping results and interpretation

Tumor profiling and serial plasma circulating tumor DNA (ctDNA) were reviewed (Table 1). The truncal drivers were an *RAF1* S257L variant, an *APC* loss-of-function variant, and a *TP53* variant—consistent with a RAS/RAF-MAPK–activated, MSS colorectal cancer.^2^  *RAF1* S257L lies at a key autoinhibitory residue; loss of inhibitory phosphorylation at this site releases RAF1 (CRAF) kinase activity and constitutively activates downstream MEK–ERK signaling. Importantly, the tumor was *KRAS/NRAS/BRAF* wild-type, so the activated MAPK signal in this tumor is attributable to the *RAF1* alteration rather than to canonical RAS/BRAF drivers.

**Table 1.** Key genomic alterations and clinical interpretation.

Alteration	Type/status	Pathway role	Clinical relevance
***RAF1* S257L**	Activating SNV; high VAF, persistent; RAF1 amplification at progression	Releases RAF1 (CRAF) autoinhibition → MAPK activation	Putative driver/target; anti-EGFR resistance mechanism
***KRAS/NRAS/BRAF***	Wild-type at baseline	Canonical MAPK drivers absent	MAPK activation attributable to RAF1; RAS-WT historically anti-EGFR eligible
**EGFR ectodomain (S492R, V441D/G, K467T)**	Acquired (progression)	Prevent cetuximab binding	Acquired anti-EGFR resistance
***KRAS* G12A, Q61H; *KRAS* amplification**	Acquired, low VAF; amplification	Reactivates MAPK downstream of EGFR	Acquired anti-EGFR resistance
***MAP2K1* (MEK1) alteration**	Acquired, low VAF	MEK-level reactivation	Possible escape from MEK/clamp inhibition
***APC*; *TP53***	Truncal LOF	WNT activation; loss of tumor suppression	Consistent with conventional CRC; not directly targetable
UGT1A1*28/*28	Germline (PGx)	Reduced SN-38 glucuronidation	Increased irinotecan toxicity; supports dose reduction
**MSI/TMB**	MSS; TMB elevated on ctDNA	—	ctDNA TMB interpretation cautious; tissue MSS argues against immunotherapy

Abbreviations: CRC, colorectal cancer; ctDNA, circulating tumor DNA; LOF, loss of function; MSI, microsatellite instability; MSS, microsatellite stable; PGx, pharmacogenomic; SNV, single-nucleotide variant; TMB, tumor mutational burden; VAF, variant allele fraction; WT, wild-type.

The molecular tumor board emphasized a key interpretive point: RAF1-driven MAPK activation is a described mechanism of resistance to anti-EGFR monotherapy.^3^ An anti-EGFR antibody alone would therefore not be expected to control this tumor, and the prior progression on panitumumab was consistent with this. This reasoning shifted the therapeutic focus from the receptor to vertical inhibition at or below the RAF node.

### Functional and clinical significance of RAF1 S257L

**Figure 1.**
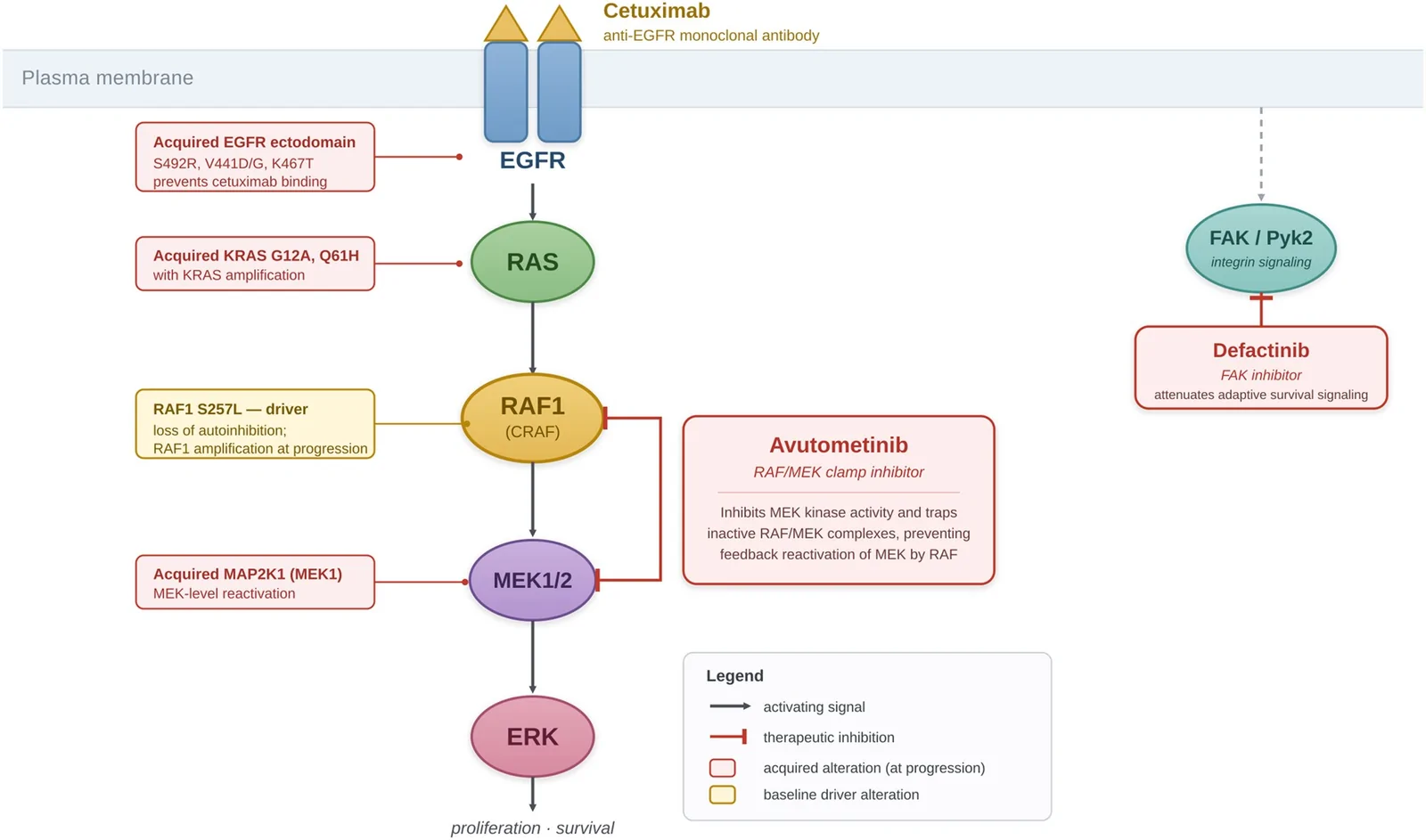
Schematic of the EGFR–RAS–RAF–MEK–ERK (MAPK) pathway in this tumor. The activating *RAF1* S257L variant releases RAF1 (CRAF) autoinhibition and drives MEK–ERK signaling. Avutometinib acts as an RAF/MEK clamp (inhibits MEK and traps inactive RAF/MEK complexes), defactinib inhibits FAK/Pyk2, and cetuximab blocks EGFR. Red labels indicate acquired alterations detected at progression (EGFR ectodomain mutations, *KRAS* alterations, *RAF1* amplification, and *MAP2K1*), reflecting convergent MAPK/EGFR reactivation. Schematic; not to scale.

RAF1 (CRAF) is one of three RAF kinases that relay signals from RAS to MEK and ERK (Figure 1). Under normal conditions, RAF1 is held inactive by intramolecular autoinhibition involving phosphorylation near serine 259^4^; the S257L substitution disrupts this autoinhibitory region, yielding ligand-independent MAPK output. In colorectal cancer, RAF1 has been shown to promote proliferation and STAT3 activation through both kinase-dependent and kinase-independent mechanisms, independent of microsatellite and *KRAS* status,^5^^,^^6^ supporting RAF1 as a biologically meaningful driver and candidate target. Because the lesion sits at the RAF node—downstream of EGFR and RAS but upstream of MEK—therapeutic logic favors inhibition at or below RAF rather than at the receptor alone.

### Potential strategies to target the pathway and implications for practice

Three vertical-inhibition strategies were considered. MEK inhibition, given evidence that MEK inhibitors can be active against RAF1-altered tumors; however, single-agent MEK inhibitors are limited by feedback reactivation of MEK by RAF. A RAF/MEK clamp inhibitor (avutometinib), which both inhibits MEK kinase activity and induces inactive RAF/MEK complexes, blunting the feedback reactivation that undermines MEK-only inhibitors; avutometinib with defactinib (a FAK inhibitor that attenuates adaptive stromal survival signaling) is FDA-approved for KRAS-mutated low-grade serous ovarian cancer^7^ and is under study with cetuximab in *KRAS*-mutated mCRC.^8^^,^^9^ Combining vertical MAPK blockade with EGFR blockade (cetuximab), with the rationale that concurrent EGFR inhibition limits upstream reactivation. The board recommended option 2 plus cetuximab, individualized to a patient who could not tolerate cytotoxic therapy or qualify for trials. The practical implication is that a rare driver, interpreted in pathway context, can support a defensible off-label regimen when standard options and trials are exhausted—provided the off-label, limited-evidence nature is explicitly discussed and consented, and intensified safety monitoring (here, weekly blood counts and a baseline electrocardiogram) is in place.

## Patient update

**Figure 2.**
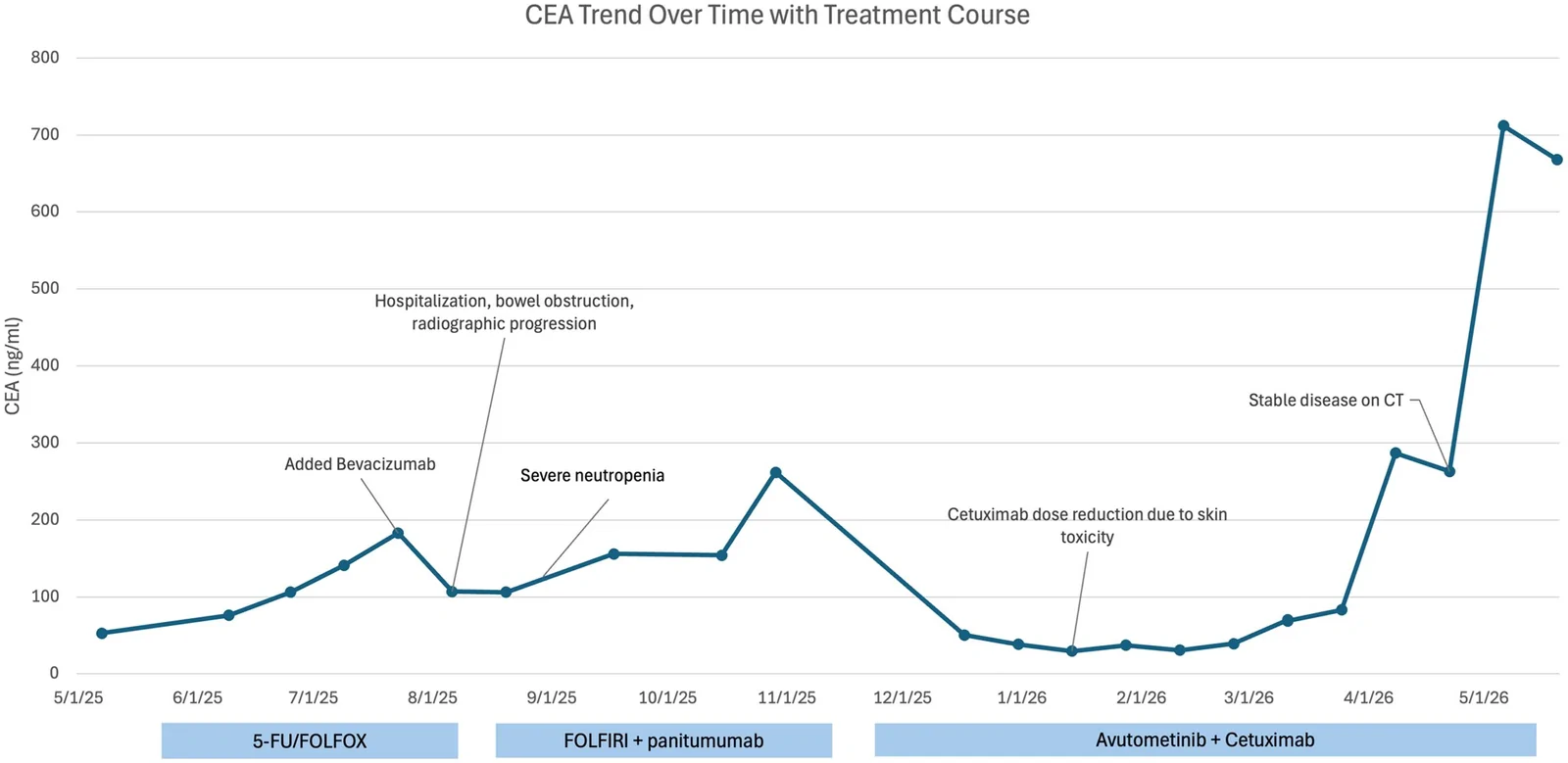
Serum CEA trend across lines of therapy (May 2025–May 2026). CEA declined during avutometinib plus cetuximab and rose at radiographic progression. Annotations mark key clinical events and the cetuximab dose reduction.

The patient began avutometinib and defactinib at standard dosing with bi-weekly cetuximab (500 mg/m^2^, later reduced by half for skin toxicity). Treatment was complicated by expected toxicity; the main adverse event was an acneiform/desquamating cetuximab-related rash with fingertip fissuring, managed in consultation with onco-dermatology with oral doxycycline and topical triamcinolone, with substantial improvement after the addition of dupilumab. Cytopenias resolved, and ECOG performance status improved from 3 to 0. Serial imaging showed regression of a right upper lobe pulmonary nodule (from 8 mm to 2 × 3 mm) with stable hepatic and peritoneal disease, and CEA declined (Figure 2). Notably, the radiographic response continued after the cetuximab dose reduction, supporting RAF/MEK clamp inhibition rather than EGFR blockade alone as the principal driver of benefit.

After approximately 6 months (10 cycles), CEA rose and CT showed progression in the liver lesion. At the time, plasma ctDNA (Table 1) showed persistence of *RAF1* S257L at high allele fraction with concurrent *RAF1* amplification, plus newly detected EGFR ectodomain mutations (including S492R), *KRAS* G12A and Q61H mutations, *KRAS* amplification, a *MAP2K1* (MEK1) alteration, and an NF1 splice-site variant, alongside *MET* and *EGFR* amplifications. This polyclonal pattern indicates convergent reactivation of MAPK/EGFR signaling—mechanistically aligned with classic acquired resistance to anti-EGFR therapy^10^—and informed selection of the next line of therapy. Together, these findings illustrate the depth of clinical benefit achievable with dual RAF/MEK clamp inhibition alongside EGFR blockade in RAF-1 mutated colorectal cancer and the eventual emergence of polyclonal, convergent resistance across the MAPK/EGFR pathway. This pattern mirrors classic anti-EGFR resistance biology and highlights the resistance mechanisms that need to be addressed in subsequent selections of therapy (Table 2).

**Table 2.** Considerations when reviewing a molecular alteration report in clinic.

Consideration	Why it matters/what to do
**Distinguish driver from passenger alterations**	Assess each alteration’s clonality and pathway role rather than treating all variants equally; a high-VAF clonal alteration in a pathway gene is more likely to be a therapeutic target than a low-VAF passenger.
**Read the alteration in pathway context**	An alteration’s actionability depends on its node (receptor vs RAS vs RAF vs MEK) and co-alterations. Vertical position guides whether receptor blockade alone is futile (eg, RAF1-driven anti-EGFR resistance).
**Check approved indication vs. tumor type**	FDA approvals are tumor- and biomarker-specific (eg, avutometinib/defactinib for KRAS-mutated low-grade serous ovarian cancer). Off-label use requires explicit rationale, consent, and documentation.
**Integrate pharmacogenomics**	Germline PGx (eg, UGT1A1*28/*28, DPYD) predicts toxicity and should modify chemotherapy dosing independent of tumor genotype.
**Account for comorbidities**	Conditions such as Gaucher disease can amplify myelosuppression and affect both regimen choice and trial eligibility.
**Use serial ctDNA to track resistance**	Acquired EGFR ectodomain and RAS alterations signal anti-EGFR resistance, often before radiographic progression; repeat testing guides the next line.
**Confirm trial eligibility early**	Cytopenias and organ dysfunction frequently exclude patients; identify this before counseling to set realistic expectations.
**Document MTB rationale and consent**	Record the evidence level, alternatives considered, monitoring plan, and shared decision-making, especially for off-label regimens.

Abbreviations: ctDNA, circulating tumor DNA; MTB, molecular tumor board; PGx, pharmacogenomic; VAF, variant allele fraction.

## Data Availability

The data supporting the findings of this study are available from the corresponding author upon reasonable request.
